# The social value of a QALY: raising the bar or barring the raise?

**DOI:** 10.1186/1472-6963-11-8

**Published:** 2011-01-11

**Authors:** Cam Donaldson, Rachel Baker, Helen Mason, Michael Jones-Lee, Emily Lancsar, John Wildman, Ian Bateman, Graham Loomes, Angela Robinson, Robert Sugden, Jose Luis Pinto Prades, Mandy Ryan, Phil Shackley, Richard Smith

**Affiliations:** 1 Yunus Centre for Social Business & Health, Research Institutes, 3rd Floor Buchanan House, Glasgow Caledonian University, Cowcaddens Road Glasgow, G4 0BA, UK; 2 Institute of Health & Society, Newcastle University, UK; 3 Newcastle University Business School, UK; 4 Centre for Social and Economic Research on the Global Environment, University of East Anglia, UK; 5 Department of Economics, University of Warwick, UK; 6 Angela Robinson, Health, Policy and Practice, University of East Anglia, UK; 7 School of Economic and Social Studies, University of East Anglia, UK; 8 Department of Economics, University Pablo de Olavide, Sevilla, Spain; 9 Health Economics Research Unit, University of Aberdeen, UK; 10 Phil Shackley, Sheffield Vascular Institute, University of Sheffield, UK; 11 Richard Smith, London School of Hygiene and Tropical Medicine, UK

## Abstract

**Background:**

Since the inception of the National Institute for Health and Clinical Excellence (NICE) in England, there have been questions about the empirical basis for the cost-per-QALY threshold used by NICE and whether QALYs gained by different beneficiaries of health care should be weighted equally. The Social Value of a QALY (SVQ) project, reported in this paper, was commissioned to address these two questions. The results of SVQ were released during a time of considerable debate about the NICE threshold, and authors with differing perspectives have drawn on the SVQ results to support their cases. As these discussions continue, and given the selective use of results by those involved, it is important, therefore, not only to present a summary overview of SVQ, but also for those who conducted the research to contribute to the debate as to its implications for NICE.

**Discussion:**

The issue of the threshold was addressed in two ways: first, by combining, via a set of models, the current UK Value of a Prevented Fatality (used in transport policy) with data on fatality age, life expectancy and age-related quality of life; and, second, via a survey designed to test the feasibility of combining respondents' answers to willingness to pay and health state utility questions to arrive at values of a QALY. Modelling resulted in values of £10,000-£70,000 per QALY. Via survey research, most methods of aggregating the data resulted in values of a QALY of £18,000-£40,000, although others resulted in implausibly high values. An additional survey, addressing the issue of weighting QALYs, used two methods, one indicating that QALYs should not be weighted and the other that greater weight could be given to QALYs gained by some groups.

**Summary:**

Although we conducted only a feasibility study and a modelling exercise, neither present compelling evidence for moving the NICE threshold up or down. Some preliminary evidence would indicate it could be moved up for some types of QALY and down for others. While many members of the public appear to be open to the possibility of using somewhat different QALY weights for different groups of beneficiaries, we do not yet have any secure evidence base for introducing such a system.

## Background

The concept of the value of a quality adjusted life year (QALY) is not new to health economics [1], but has reached prominence in policy and in empirical research due to the creation of national-level health technology assessment agencies [2-5]. When assessing particular interventions in terms of health gains against the costs of provision, such agencies must, in effect, put a monetary value on those health gains. In the context of England, where the National Institute for Health and Clinical Excellence (NICE) uses the QALY as its health metric, NICE must decide what value(s) of a QALY to use.

Since the inception of NICE, the threshold value of a QALY has been £20-30,000 [6]. Interventions with a cost per QALY above this range are less likely to be recommended by NICE for adoption by the rest of the NHS [7,8]. The threshold was based on best guesses of experts at the inception of NICE^6 ^and has been subject to criticism since the UK House of Commons Health Committee review of NICE in 2001-2 [9]. The criticism at that time centred on the lack of an empirical basis for the threshold. More recently, pressure has been placed on NICE to raise the threshold, as exemplified in the case of life-extending drugs for people in the terminal phase of cancer [10,11], and to lower it, based on forthcoming fiscal pressures and views of primary care trusts (PCTs) that NICE guidance is not affordable [12]. Indeed, arguments for and against raising the threshold were debated in a recent *head-to-head *in the *BMJ *[13,14].

Partly in response to such criticism, NICE co-funded two research projects in 2004. One of these, the Social Value of a QALY (SVQ) project, was conducted by the authors of this paper. The results of SVQ have recently been published [15,16], and used in some quarters to put further upward pressure on the threshold [10,11,13,17]. However, use of the results in such reporting has been selective. It is important that a summary overview of the project is presented. Also, the views of those who conducted the research as to its implications for the NICE threshold have not been expressed before. The purposes of this paper are to provide such a summary and interpretation for policy. Of course, in making a summary and drawing interpretations, it is important to recognise that, as with all such valuation approaches, the methods used are controversial. The reader is able to draw conclusions on such issues from reading more in-depth reports of these methods in other peer-reviewed publications [15,16]. Nevertheless, returning to the main point of this paper, it will be seen that the issue of whether to raise the value-of-a-QALY bar or bar the raise is somewhat more complex than discussed to date and that both sides in the debate are right, but also wrong.

## Discussion

### The SVQ project

There are different ways of seeking a value of a QALY. Valuable information has been generated, for example, in analysis of the affordability and cost of generating a QALY at the level of PCTs, given their pre-determined budgets [18,19]. SVQ consisted of three related strands, each based on eliciting values from members of the general public. The first strand involved modelling the monetary value of a QALY from the willingness-to-pay (WTP)-based value of preventing a statistical fatality (VPF) that the UK Department for Transport (DfT) and other public sector agencies apply to life-saving projects. This value is derived from asking representative samples of the public about their WTP for safety improvements. These improvements are characterised as reducing the risk of death for any individual by small amounts in the forthcoming time period (e.g. the coming year). Across a population, a small number of actual lives will be saved. In simple terms, dividing aggregate WTP of the population by this small number of lives saved gives us the VPF. With WTP values being elicited from a cross-section of the population it can be argued that the resulting VPF (or value of a QALY, if that is the focus of interest) is reflective of society's overall budget constraint. An important ethical standpoint is that the resulting 'average' value is applied to each member of society regardless of income. Indeed, public sector agencies that employ WTP-based values (such as the DfT and the Health and Safety Executive in the UK) invariably do apply the *same *value, based on the population *average*, to all income groups.

A simplified version of the method of transforming the VPF into a value of a QALY is as follows:

A straightforward way to compute the value of a QALY is to start with the well-established roads VPF for the UK. For example, if we take a representative death avoided as being that of a person aged 35, assume that the VPF is £1.4 m (or £1.4 × 10^6^) and that the person concerned would have lived for another 40 years, a rough calculation of the value of a life year gained by that person would be as follows:

$$\begin{array}{c} \text{V}=\frac{\text{£1}\text{.4}\times {\text{10}}^{6}}{40}\\ \;=\;£\text{35},000 \end{array}$$

Conveniently, V is close to the value of a QALY espoused by Rawlins and Culyer in their 2004 *British Medical Journal *paper [6]. However, if one were to assume that not all of the 40 years gained would be spent in full health (especially later years) and a discount rate applied, the denominator would fall, thus raising the value of a QALY above £35,000. For example, if the discount rate was taken to be 3.5% then the annualised sum that would have a discounted present value of £1.4 m over 40 years would be £77,300.

However, the value resulting from this would reflect a particular QALY type. By QALY-type, we mean that QALYs can be generated in at least two ways; these being by adding years to life or by enhancing the quality of remaining life years without extending life. The former can be further subdivided into avoiding immediate threats to life or adding years to the end of one's life. The procedure outlined above reflects the first of these, although a more-sophisticated approach, still using the VPF was also used to model the value of a QALY arising from life extension as opposed to life saving [15]. Similar WTP procedures were also used by DfT to derive the VSI, or value of a serious injury prevented [20], from which a value of a quality-of-life-enhancing QALY can be derived [15], summarised as follows:

Each serious injury was broken down into 3 phases; in hospital effect (valued at 0.69 or 0.16 on the EQ-5D tariff, depending on severity of injury and generally modelled as lasting for one month), initial after-effects (generally for two months and valued at 0.76) and longer-term effects (for remaining life and valued at either 0.76 or 0.3, again depending on severity). Assuming that any given injury would occur at the mean age of the UK population, with 26 expected remaining QALYS, we calculated an overall total QALY loss for each scenario. We then divided the VSI of £150,000 by the total QALY loss for each scenario and computed a weighted average based on probability of each scenario occurring.

Thus, values for all three QALY types could be explored within the research.

Note that, beyond this, SVQ did not look at the value attached to QALY gains from treating specific diseases. This is due to a more generic rather than disease-specific approach to economic evaluation being the tradition in UK health economics and decision making.

The second strand of SVQ assessed the feasibility of obtaining an estimate of the monetary value of a QALY by presenting members of the public with appropriately framed valuation questions in a survey. Example health states are as follows:

Stomach: 3 months

Initially you will have severe stomach pains, diarrhoea, vomiting and fever for 7 days, severe enough to interfere with most of your usual activities.

Things then improve, but for up to one year from initial onset you will suffer an episode of stomach discomfort and sickness every couple of weeks, with each episode lasting for 2-3 days. These episodes are not so severe but may interfere with some of your usual activities.

(*Half of the respondents were given stomach health state descriptions of 3 months, 12 months and lifetime durations*.)

Head: 3 months

You will have episodes of throbbing pain across the front of your head and you will feel sick and may occasionally be sick. You will feel like you want to lie still in a darkened room.

During the next three months you will suffer an episode of head pain and sickness every couple of weeks, with each episode lasting between 8 hours and two days. These episodes will interfere with many of your usual activities. After three months you return to your current health with no further effects from this illness.

(*The other half of the respondents were given head health state descriptions of 3 months, 12 months and lifetime durations*.)

An example question to illustrate how changes in quality of life and WTP were estimated and combined was as follows:

The value of a QALY is derived via a 'chaining' procedure. In the initial part of the chain, the respondent is asked about whether s/he would be prepared to pay anything to avoid being in this state, and, if so, what is the maximum amount s/he is willing to pay.

In the second part of the chain, the respondent would be asked a 'standard gamble' question involving a choice between two options. In the standard way of deriving a QALY index, one option would leave the respondent in the stomach/head condition for certain for the remainder of his/her life whilst the other option would involve a gamble with varying probabilities of a better or worse outcome. 'Better' usually means a return to full health for the rest of one's life, whilst worse is usually characterised as immediate death. Visual procedures are used to guide the respondent through the process, and the index is derived from the point at which the respondent feels it is difficult to choose between the outcome for certain and the gamble.

Let us assume that, for one respondent, the probability at which s/he finds it difficult to choose between the head condition for certain and taking the gamble is 0.95 and that his/her WTP to avoid a year in the stomach condition was £1000. Dividing £1000 by 0.05 (which comes from subtracting 0.95 from 1) would give a value of a QALY for that person of £20,000. This can be done across several individuals to arrive at an average value of a QALY for a population.

For either head or stomach conditions, each respondent was asked two WTP questions (to avoid the three-month state and the 12-month state) and three standard gamble questions (3 months for certain vs a gamble with outcomes of return to current health or 12 months in the state; 12 months for certain vs a gamble with outcome of return to current health or rest of life in the state; and rest if life for certain vs gamble with outcomes of current health or immediate death). In fact slightly more WTP and standard gamble questions were asked of each respondent, but these are not relevant to this paper.

From this, it can be seen that any individual respondent would be faced with a set of WTP and standard gamble questions, the two sets then being combined in different ways to arrive at values of a QALY. Respondents could have been asked time trade-off questions instead of standard gambles, our rationale for the latter simply being that it comes from the same theoretical 'stable' as WTP. On the other hand, given that the QALY tariff used by NICE is based on time trade-off (using 10-year time horizons) it is not necessarily the case that direct comparison between the value of a QALY derived from SVQ and NICE's valuation of a QALY can be made. Also, rather than combine WTP values with a pre-existing tariff (such as that which exists for the EQ-5D quality of life system), we wanted our own respondents to provide health state utility values that could be combined with their own WTP values for purposes of internal consistency. Eliciting WTP from our sample and then combining these with EQ-5D values from a different population would also have been problematic. Of course, standard gambles are known to have problems with lack of sensitivity [21], and WTP methods, as with other valuation methods, have been robustly criticised and defended, and are thus controversial [22]. However, the aim of this part of SVQ was to test the feasibility and robustness of the elicitation methods; and, rather than conduct a full-scale national sample survey, the sample was restricted to 400 people which was not representative of the population.

The third strand aimed to investigate the extent to which members of the public consider that there may be legitimate grounds for distinguishing between the social value to be accorded to QALYs gained by different types of patient. It should be noted here that, rather than being about the personal (or individual) value of a QALY, as in strands 1 and 2, strand 3 is about valuing QALYs in others. For example, should a particular QALY gain delivered to someone currently in very poor health be weighted differently from the same number of QALYs delivered to people whose current health state is not nearly so bad? Should a paediatric QALY be weighted differently from a geriatric QALY? As in the disability adjusted life year literature, should gains to the very young and older people be given less weight than those of productive age? [23] Descriptions of the two main types of question used in SVQ to examine these issues are as follows:

Person trade-off (PTO)

Using this method, respondents in SVQ were asked how many people of certain characteristics (e.g. in terms of stage of life and/or severity of condition) and achieving some sort of QALY gain (usually four QALYs) would be judged equivalent to, say, 100 people with different degrees of the same characteristics who might also achieve such a gain. If a number less than 100 is given in response that would mean that the gains to the former group are valued higher than to the latter. A series of such questions can be asked to try to establish the exact person trade off (where the respondent cannot choose between the two options). These numbers can then be used to establish what the 'weights' for different scenarios presented would be for one individual and can be aggregated to establish what the weights might be at the population level.

Discrete choice experiments (DCEs)

Using this method in SVQ, respondents were presented with a series of choices between scenarios. These scenarios looked similar to those used in the PTO with choice made on the basis of stage of life and severity, except that (a) the size of the health gain was also allowed to vary across the scenarios in any one choice set and (b) the respondent merely chose one scenario in preference to the other, and did not use numbers of people to weight the scenarios. Again, with each respondent making a series of such choices, statistical procedures can be employed to arrive at a set of weights across a population sample.

(*Each respondent answered 6 PTO and 8 DCE questions. To aid the process, these questions were presented in a diagrammatic form in a computer-assisted version of the questionnaire. This procedure was developed in over a year of developmental and piloting work prior to the main survey*.)

The person trade-off method was based on the earlier work on Nord [24,25] and the discrete choice methods were new to this area of application.

### QALY-types

Table 1 gives a typical set of values of a QALY that have arisen from the modelling. It would seem that different 'QALY-types' would imply different values. Based on WTP to reduce the risks of life-threatening events, values close to £70,000 per QALY were produced, as compared to values around £35,000 for a life-extending QALY. Estimating gains from improvements in quality of life, with no increase in number of remaining years, produced a lower value of about £10,000 per QALY.

**Table 1:** Values of a QALY via alternative calculations from modelling based on VPF and VSI

Basic modelling approach	Value of a QALY (£)
Life-saving	£70,000

Life-extending	£35,000

Quality-of-life-enhancing	£10,000

Nevertheless, it is worth noting that the above results imply that the threshold could be raised for life-saving QALYs. One note of caution, however, involves reflecting on what might be meant by a life-saving QALY being valued at seven times that of a quality-of-life-enhancing QALY. Although not calculated like this, it might be reasonable to assume that the 'average' utility score across the profiles of serious injuries were, say, 0.6. This would imply that WTP to avoid a fatality would be just over 11 times that for the serious injury (i.e. 70,000/10,000 × 1.0/0.6). This, along with the results in Table 1 therefore, presents a hypothesis that requires further testing. (It is worth noting here that the issue of WTP for QALY types was not explored directly in survey work in SVQ but has been incorporated into subsequent surveys on the 'European value of a QALY' - see the EuroVaQ website at http://research.ncl.ac.uk/eurovaq/). It is also worth noting that the VPF itself is just over nine times the value of the VSI. That there is no single value of a QALY is in line with other published views [26], the lowest value also being reflective of earlier published studies which looked at the value of QALY gains arising from quality-of-life enhancement only [3,27].

But where does this leave particular groups, such as those with very low remaining life expectancy who will die prematurely anyway (such as cancer patients "close to death")? SVQ does not have a specific answer to this, as life-saving QALY values arising from the project are based on the assumption that those saved will go on to live a full and healthy life. The analytical framework applied in the first strand of SVQ (and developed further in a related study of nuclear risks commissioned by the Health and Safety Executive [28]) suggests that there ***might ***be a case for higher values to be assigned to QALYs delivered to those categories of patient. However, this case would be based on the values of people close to death only. Drawing parallels with the earlier argument (in the Methods section) regarding income groups, this would contravene an ethical standpoint supporting the application of an average value from a cross-section of the population to all members of that population.

### Results of fieldwork investigating the value(s) assigned to a QALY

The second strand of the study suggested that it is feasible to conduct a survey to elicit monetary values for a QALY from a representative sample of the public so long as the procedure is broken down into manageable steps and is carried out on a face-to-face basis by well-trained interviewers. However, it also became apparent that the mean estimates produced by such questions are particularly prone to the influence of "outlier responses" and that great care is therefore required in the selection of central-tendency measures. The most common example of an outlier was that many people were willing to take only very small risks of a more adverse outcome to avoid the stomach and head health states in the standard gamble questions, or were even not willing to gamble at all. As well as such floor effects, respondents may also have a WTP ceiling (or budget constraint), an amount they express whether for a small or large perceived gain. Thus, when WTP values and health state utilities are combined in such circumstances, the implied WTP per QALY for such individuals can be so high as to lead to an implausible population average WTP per QALY across the whole sample. This was indeed the case in SVQ, with the value running into several millions of pounds!

Other ways of managing the data, therefore, are displayed in Table 2. Rather than computing a ratio of WTP/QALY loss for each individual and then taking a mean, the first two calculations take the mean WTP, the mean QALY loss and then compute the ratio. This is done for each of the stomach and head scenarios. The third calculation takes a ratio of medians. So, for example, using median stated WTP to avoid the certainty of a 12-month period of illness, the figures suggest a value for a QALY in the region of £20,000-£40,000.

**Table 2:** Values of a QALY via calculations from survey research

Using 12-month (mean) WTP value and (mean) standard QALY index:		
**CONDITION**	**CALCULATION**	**VALUE OF QALY**

	1. Mean WTP to avoid 12-month S = £1870	
Stomach	2. Average QALY benefit = 0.104	£17,980
	Divide 1. by 2.	

	1. Mean WTP to avoid 12-month H = £3250	
Head	2. Average QALY benefit = 0.144	£22,570
	Divide 1. by 2.	

Using 3-month (mean) WTP value and (mean) standard QALY index:		

CONDITION	CALCULATION	VALUE OF QALY

	1. Mean WTP to avoid 3-month S = £810	
Stomach	2. Average QALY benefit = 0.026	£31,150
	Divide 1. by 2.	

	1. Mean WTP to avoid 3-month H = £1495	
Head	2. Average QALY benefit = 0.036	£41,530
	Divide 1. by 2.	

Using 12-month (median) WTP value and (median) standard QALY index:		

CONDITION	CALCULATION	VALUE OF QALY

	1. Median WTP to avoid 12-month S = £500	
Stomach	2. Median QALY benefit = 0.025	£20,000
	Divide 1. by 2.	

	1. Median WTP avoid 12-month H = £1000	
Head	2. Median QALY benefit = 0.025	£40,000
	Divide 1. by 2.	

Finally, in the third strand of the SVQ study, aimed at quantifying the effect of age and health status on the public's valuation of QALY gains, as is often the case where two different approaches are used, each produced somewhat different results. This discrepancy between the results of the two approaches reflects the differing results based on similar approaches that is emerging over time in the literature [29-32], and will almost certainly require further research if a definitive resolution is to be established. Nevertheless, one of the approaches appeared to provide grounds for giving significantly greater weight to the value of QALYs gained by younger adults suffering from fairly severe health impairments as compared with very small children or elderly people, especially if their impairments were not very severe.

### Further research

Given the degree of variability in values reported, the issue of differential values for QALY types requires urgent attention. Three issues are particularly worthy of this. In chaining the values derived from standard gamble and WTP questions there appears to be a methodological problem in identifying health states which are serious enough to encourage more respondents to trade them (against risks of death and full health) in standard gambles, but which are not so serious that paying for their avoidance is perceived as unaffordable in WTP questions.

The second issue is that of the value of 'QALY types'. Initial evidence from the first strand of the research reported indicates that different types of QALYs may have different values. The results in Table 1 indicating this require further research in order to be confirmed or refuted. Linked to this, a major policy gap also seems to be that of providing valuations to be attached to short-term QALY gains for those in terminal phases of illness. Notably, NICE has raised the threshold for such conditions; although this would be expressed by them as a weighting of the single £20,000-£30,000 threshold rather than having changed the threshold as such.

Thirdly, at least some of the study's findings suggest that differential weighting of QALY gains by characteristics of beneficiaries is a possibility deserving further consideration. In terms of research, the two approaches used in SVQ (discrete choice and PTO) are worthy of refinement and comparison. Furthermore, although not explicitly researched in SVQ, the concern of people for ensuring the right to realise health potential [33,34] could be incorporated into such future work. This concern is based on the egalitarian view that people be allowed to realise their potential for health and that rights to access care should depend less on maximising gains in quality or length of life. Evidence that this is of concern to the public has been found in several countries [33-36], including the UK [37], and would also explain somewhat the position of other HTA agencies, such as the Institute for Quality and Economizing in Health Care (IQWiG) in Germany, to use disease-specific outcome measures within different therapeutic areas. IQWiG's position could be interpreted as meaning that patient groups in which treatment is highly effective relative to resources expended should not necessarily take priority over groups where treatment outcomes for resources used are more modest [38].

## Summary

On the question of whether it is feasible to estimate a monetary value of a QALY, the jury is still out: probably the most that can be said is that, based on population average values derived from survey research, there is as yet no compelling evidence for moving the current threshold either up or down. Although ours was merely a feasibility study, it would be reasonable to say that the NICE response to the work conducted in SVQ was one of reassurance over this position.

Nevertheless, raising the threshold for some conditions should not necessarily be ruled out on grounds of affordability. Rarer and higher-valued (life-saving) QALYs (such as for heroic, or even some routine, types of surgery) could be paid for through having a lower threshold for more common quality-of-life-enhancing types of QALY. This is what we mean by both sides of the threshold-level debate being right and wrong, although such a move would require in-depth validation of the results on QALY types displayed in Table 1.

While many members of the public appear to be open to the possibility of using somewhat different QALY weights for different groups of beneficiaries, we do not yet have any secure evidence base for introducing such a system. In England, this remains reflected in the recently-revised social value judgment document produced by NICE, published after the submission of the report of the SVQ project [39].

## List of abbreviations

DfT: Department for Transport; PTO: Person trade-off; QALY: Quality adjusted life year; NHS: National Health Service; NICE: National Institute for Health and Clinical Excellence; SVQ: Social Value of a QALY; VPF: Value of a prevented fatality; VSI: Value of a serious injury; WTP: Willingness to pay

## Competing interests

The authors declare that they have no competing interests.

## Authors' contributions

CD was principal investigator and made substantial contributions to the conception of the overall study as well as the design of each of the three major components and to writing; RB made substantial contributions to the conception of the overall study as well as the design of each of the three major components and to writing; HM designed and led the analysis of the work on modelling the value of a QALY and contributed to the design of each of the three major components and to writing; MJ-L made substantial contributions to the conception of the overall study as well as the design of each of the three major components and to writing: EL made substantial contributions to discrete choice method used in the study and to writing; JW led the analysis of discrete choice data and contributed to writing; IB contributed to the conception of the overall study as well as the design of each of the monetary valuation survey and the two methods used to derive QALY weights; GL, as co-PI, made substantial contributions to the conception of the overall study as well as the design of each of the three major components, analysis of the monetary valuation data and to writing: AR made substantial contributions to the conception of the overall study as well as the design of each of the three major components and to writing; RS contributed to the design of each of the methods used to derive QALY weights and to writing; JLPP contributed to the design of the monetary valuation survey; MR contributed to the design of each of the discrete choice method and to writing; PS contributed to the conception of the overall study as well as the design of the monetary valuation survey and the survey on QALY weights; RS contributed to the conception of the overall study as well as the design of the monetary valuation survey and the survey on QALY weights. All authors read and approved the final manuscript.

## Pre-publication history

The pre-publication history for this paper can be accessed here:

http://www.biomedcentral.com/1472-6963/11/8/prepub
